# Metastatic Patterns of Myxoid/Round Cell Liposarcoma: A Review of a 25-Year Experience

**DOI:** 10.1155/2012/345161

**Published:** 2012-04-01

**Authors:** Naofumi Asano, Michiro Susa, Seiichi Hosaka, Robert Nakayama, Eisuke Kobayashi, Katsuhito Takeuchi, Keisuke Horiuchi, Yoshihisa Suzuki, Ukei Anazawa, Makio Mukai, Yoshiaki Toyama, Hiroo Yabe, Hideo Morioka

**Affiliations:** ^1^Department of Orthopaedic Surgery, Keio University School of Medicine, 35 Shinanomachi, Shinjuku-ku, Tokyo 160-8582, Japan; ^2^Department of Orthopaedic Oncology, Tochigi Cancer Center, 4-9-13 Younann, Utsunomiya-shi, Tochigi 320-0834, Japan; ^3^Department of Orthopaedic Surgery, Kyosai Tachikawa Hospital, 4-2-22 Nishikicho, Tachikawa-shi, Tokyo 190-8531, Japan; ^4^Department of Orthopaedic Surgery, Tokyo Dental College Ichikawa General Hospital, 5-11-13 Sugano, Ichikawa-shi, Chiba 272-8513, Japan; ^5^Department of Diagnostic Pathology, Keio University School of Medicine, Tokyo 160-8582, Japan

## Abstract

Myxoid/round cell liposarcoma (MRCL), unlike other soft tissue sarcomas, has been associated with unusual pattern of metastasis to extrapulmonary sites. In an attempt to elucidate the clinical features of MRCL with metastatic lesions, 58 cases, from the medical database of Keio University Hospital were used for the evaluation. 47 patients (81%) had no metastases, whereas 11 patients (11%) had metastases during their clinical course. Among the 11 patients with metastatic lesions, 8 patients (73%) had extrapulmonary metastases and 3 patients (27%) had pulmonary metastases. Patients were further divided into three groups; without metastasis, with extrapulmonary metastasis, and with pulmonary metastasis. When the metastatic patterns were stratified according to tumor size, there was statistical significance between the three groups (*P* = 0.028). The 8 cases with extrapulmonary metastases were all larger than 10 cm. Similarly, histological grading had a significant impact on metastatic patterns (*P* = 0.027). 3 cases with pulmonary metastatic lesions were all diagnosed as high grade. In conclusion, large size and low histological grade were significantly associated with extrapulmonary metastasis.

## 1. Introduction

 Liposarcoma is one of the most common subtypes among soft tissue sarcomas constituting 9–18% of all cases [1]. The incidence peaks between the 4th and 5th decades of life and there is a slight male predominance. The World Health Organization recognizes four subtypes: well differentiated, dedifferentiated, myxoid/round cell, and pleomorphic. The myxoid/round cell subtype is among the most prevalent and typically occurs in younger individuals [2]. It characteristically occurs in the deep-seated soft tissues of the extremities, especially in the thigh which comprises two-thirds of the group.

Another important distinction from other types of liposarcomas is that MRCL is associated with an unusual pattern of metastasis to bone such as spine and other soft tissues such as retroperitoneum, limb, and axilla [2, 3]. Other soft tissue sarcomas tend to metastasizes to the lung, while other sites are typically involved in advanced stages of the disease [2, 3]. Although the predilection of MRCL for extrapulmonary metastases is widely accepted, there is no data regarding which MRCL metastasizes to the lung and which MRCL metastasizes to extrapulmonary sites. The purpose of this study is to elucidate the clinical features of MRCL with metastatic lesions.

## 2. Materials and Methods

### 2.1. Patients

We evaluated 58 cases of MRCL from the medical database of Keio University Hospital between 1985 and 2010. Median followup was 78 months (range; 3–290 months). All primary tumors were imaged with MRI. All but one primary tumor were managed by surgical treatment. Radiotherapy and chemotherapy were utilized on a case-by-case basis. All patients were followed clinically and with radiographic surveillance (chest X-ray and/or CT) for metastasis. Local recurrence was monitored by physical examination and MRI. Additional screening tests, ordered at the discretion of the physician to rule out extrapulmonary metastasis, included bone scintigraphy, CT of abdomen/pelvis, and MRI of the spine.

### 2.2. Definitions

In order to determine the clinicopathological features of metastasis in MRCL, we divided patients into three groups; (1) without metastasis, (2) with extrapulmonary metastasis, and (3) with pulmonary metastasis. If patients had both pulmonary and nonpulmonary metastases during clinical courses, we grouped them according to their first metastasis site.

**Figure 1:**
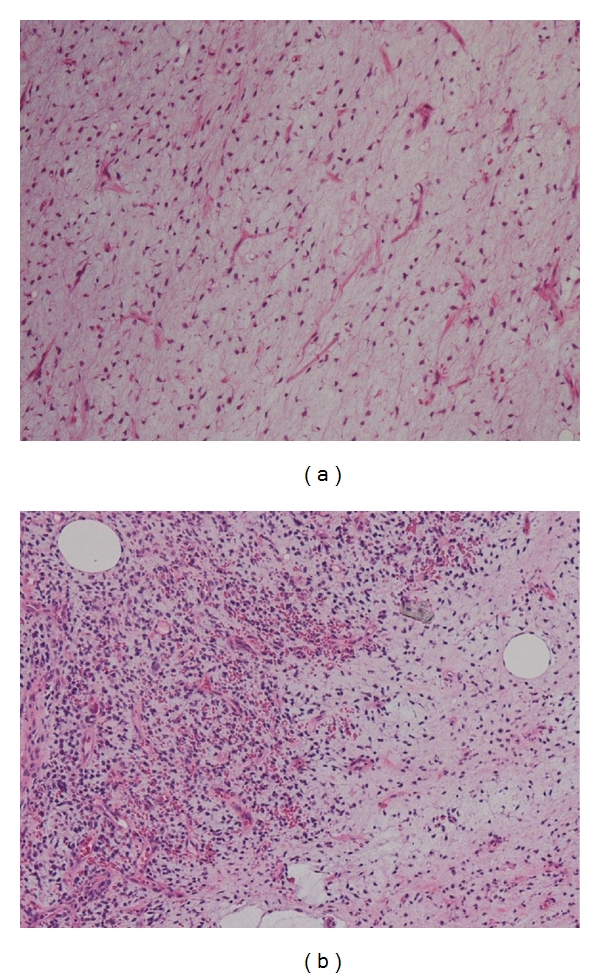
(a) Photomicrograph of low-grade MRCL. (b) Photomicrograph of high-grade MRCL.

Tumors demonstrating a round cell component of less than 5% of tumor volume were designated as low grade (Figure 1(a)) and tumors with round cell component of higher than 5% of tumor volume were considered high grade (Figure 1(b)) as described previously [4]. The percentage of round cells was estimated by scanning all individual sections (average 1 block/1 cm^3^ of tumor) of the resected specimen. The percentage of round cell component was estimated by scanning all individual sections, using the entire tumor volume as a denominator as described previously by other authors [4, 5].

### 2.3. Demographics

Of the 58 patients with MRCL identified from our sarcoma database, 33 were male and 25 were female. Median age at presentation was 46 years old (range; 18–80 years). 19 patients (33%) had tumors greater than 10 cm in size. 39 of 52 patients (75%) had tumors beneath the subcutaneous layer. 48 patients (83%) had the primary tumor in the extremities and 10 (17%) in the trunk. The most common site was the thigh occurring in 30 patients, followed by lower leg in 9 patients, upper arm in 4 patients, retroperitoneum in 2 patients, and 1 each of shoulder, elbow, forearm, popliteal, foot, chest wall, groin, lower back, and buttock.

### 2.4. Histology

38 patients (66%) were diagnosed as low grade MRCL, whereas the remaining 20 cases (34%) with more than 5% of round cell morphology were diagnosed as high grade MRCL. Within low grade MRCL, 21 patients (36%) had pure myxoid component and 17 patients (29%) had less than 5% of round cell area.

### 2.5. Operative Treatment

All but one patient underwent surgical resections. Wide or radical resection was achieved in 40 patients (69%), whereas the remaining patients had marginal (16 patients, 28%) or intralesional resections (one patient, 13%).

### 2.6. Chemotherapy

Twenty-nine (50%) of 58 MRCL patients received variable chemotherapy for the primary tumors: 3 as neoadjuvant therapy, 21 as adjuvant therapy, 5 as neoadjuvant and adjuvant therapy. The details of regimens were available in 25 patients. Treatments were highly heterogeneous. 5 different regimens were administered with doxorubicin-containing regimens comprising the largest subgroup; doxorubicin + ifosfamide: total 17 courses in 11 patients, doxorubicin + cisplatin/ifosfamide + etoposide: total 18 courses in 10 patients, vincristine + doxorubicin + cyclophosphamide/ifosfamide + etoposide: total 4 courses in 2 patients. The remaining regimens were comprised of conventional or experimental agents; cisplatin + etoposide: 3 courses in 2 patients, ifosfamide: 2 courses in one patient. Response data were not available.

### 2.7. Radiotherapy

We performed external radiation therapy for the high-risk cases such as close surgical margin or huge tumor size. Thirteen (22%) of 58 MRCL patients received radiotherapy to the primary lesions: 2 as neoadjuvant (40 Gy) and 11 as adjuvant (50–68 Gy).

### 2.8. Data

Age, gender, tumor location, size, depth, histological grade (low versus high), surgical margin, addition of radiotherapy (yes versus no), addition of chemotherapy (yes versus no), local recurrence, and clinical outcome were analyzed for its correlation to metastasis. We designated initial time as the date of initial diagnosis of MRCL and final time as the date of last visit.

### 2.9. Statistical Analysis

Categorical data were analyzed with chi-square test (William's correction) using StatMate IV (ATMS Co. Ltd., Tokyo, Japan). Survival data were calculated using Kaplan-Meier method. Results with *P* < 0.05 were considered statistically significant.

## 3. Results

47 patients (81%) had no metastases, whereas 11 patients (19%) had metastases during their clinical course. Local recurrence occurred in 5 patients (9%). All local recurrences were resected with wide margins. Among the 11 patients with metastatic lesions, 8 patients (73%) had extrapulmonary metastases and 3 (27%) had pulmonary metastases. All 8 patients with extrapulmonary metastasis had no pulmonary metastases during their clinical course. One of 3 patients with pulmonary metastasis experienced both pulmonary and extrapulmonary metastasis.

**Figure 2:**
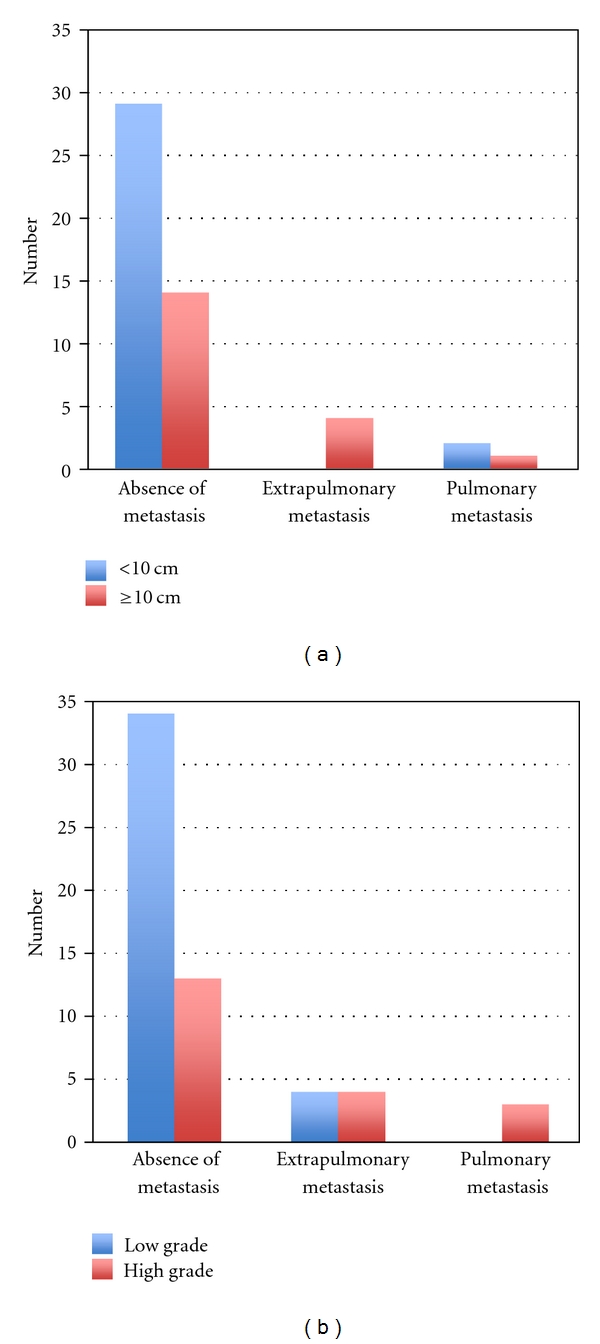
(a) Correlation of metastatic pattern to tumor size. When the metastatic patterns were stratified according to tumor size (<10 cm or ≥10 cm), there was statistical significance between the groups (*P* = 0.028). Of the 47 cases without metastases, 14 (38%) were larger than 10 cm. The 8 cases with extrapulmonary metastases were all larger than 10 cm. Only 1 case was larger than 10 cm in the pulmonary metastatic group. (b) Correlation of metastatic pattern to histological grade. Histological grading (low or high) had a significant impact on metastasis patterns (*P* = 0.027). Of the 8 cases with extrapulmonary metastases, 4 (50%) were pathologically diagnosed as low-grade MRCL and 4 (50%) as high-grade MRCL. The 3 cases with pulmonary metastatic lesions were all diagnosed as high-grade MRCL.

**Figure 3:**
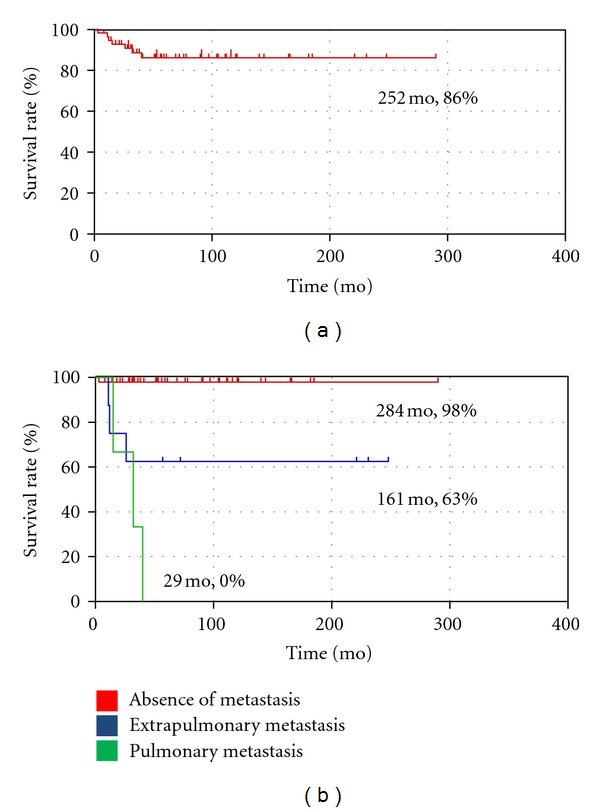
Survival rate. (a) The absolute overall survival rate was 86%. (b) The overall survival rate was significantly better for patients with extrapulmonary metastasis (63%) compared to those with pulmonary metastasis (0%) (*P* < 0.001).

When the metastatic patterns were stratified according to tumor size (<10 cm or ≥10 cm), there was statistical significance between the three groups (*P* = 0.028) (Table 1). Of the 47 cases without metastasis, 14 (38%) were larger than 10 cm. The 8 cases with extrapulmonary metastases were all larger than 10 cm, whereas only 1 case (33%) was larger than 10 cm in pulmonary metastatic group (Figure 2(a)). Similarly, histological grading had a significant impact on metastasis patterns (*P* = 0.027) (Table 1). Of the 8 cases with extrapulmonary metastases, 4 (50%) were pathologically diagnosed as high grade MRCL, whereas 3 cases with pulmonary metastases were all diagnosed as high grade MRCL (Figure 2(b)). Surgical margin, radiotherapy, and local recurrence had no significant effect on metastatic patterns (Table 2). Chemotherapy was performed in 20 cases (43%) without metastases, 6 cases (75%) with extrapulmonary metastases, and 3 cases (100%) with pulmonary metastases. Chemotherapy was not effective in reducing disease-free survival rate, but implementation of chemotherapy had statistically significant influence on metastatic patterns (*P* = 0.003). Of the 58 patients, 43 were continuously disease free (CDF), 7 had no evidence of disease (NED), 1 was alive with disease (AWD), and 7 were dead of disease (DOD). In 8 patients with extrapulmonary metastases, 4 (50%) were NED, 1 was AWD, and 3 (38%) were DOD. There was no survivor with pulmonary metastases (*P* < 0.001) (Table 2). Duration from primary surgery to first metastasis was longer in patients with extrapulmonary metastases (median 55 months) compared to patients with pulmonary metastases (median 16 months) (*P* = 0.069). The absolute overall survival rate was 86% (Figure 3(a)). The overall survival rate was significantly better for patients with extrapulmonary metastases (63%) compared to those with pulmonary metastases (0%) (Figure 3(b)).

## 4. Discussion

In contrast to other soft tissue sarcomas which metastasize primarily to the lung, MRCL is associated with an unusual pattern of metastasis. Previous reports have shown metastases of MRCL to extrapulmonary sites, including the retroperitoneum, subcutaneous soft tissue and bone [2, 3, 5, 6]. Antonescu and Blair reported that MRCL in particular tends to spread to other soft tissue sites including retroperitoneum, thorax, and extremity before metastasizing to the lung [4, 5, 7]. Also, in the previous large series of MRCL, extrapulmonary metastatic rate in MRCL was 17–30% and common sites of extrapulmonary metastases were bone, soft tissue of extremity, retroperitoneum, abdomen, and chest wall [2, 6, 8, 9]. Skeletal metastasis has recently been reported as the most common site of metastasis in MRCL [2]. Schwab et al. identified 8 patients with skeletal metastases on radiographic findings in a population of 184 patients with MRCL, an incidence of 4.3% [2]. Schwab et al. reported that more than half (56%) of the total metastatic sites represent skeletal metastases, 70% in the absence of pulmonary spread, and a high incidence of metastasis to the spine [2]. 

MRCL contains the specific *t* (12; 16) chromosomal translocation, which results in rearrangement of the *TLS* and *CHOP* genes that is clone specific at DNA level [10–12]. Three common forms of the *TLS-CHOP* fusion have been described, differing by the presence or absence of *TLS* exons 6–8 in the fusion product. Type I includes *TLS* exon 6 and 7 in the fusion, type II consists of *TLS* exon 1–5 fused to *CHOP* exon 2, and type III fuses *TLS* exons 1–8 to *CHOP* exon 2 [5]. A specific *TLS-CHOP* fusion gene resulting from the *t* (12; 16) is present in at least 95% of MRCL [5]. 85% of the patients who developed bone metastases showed a type II *TLS-CHOP* fusion transcript [2]. Ogose et al. reported that the reason for the high incidence of extrapulmonary metastases in MRCL is unclear, but an abundance of fat cells in metastatic sites, such as subcutaneous tissue, retroperitoneum, bone marrow, and epidural space, may contribute to the high incidence of these unusual metastases [13]. In our study, extrapulmonary metastases rate in MRCL was 14%. Common locations for extrapulmonary metastases were bone and soft tissue of extremities, similar to the previous studies (Table 3).

MRCL patients have been reported to present more commonly with multifocal disease, either synchronous or metachronous, compared to other soft tissue sarcomas [7]. Multifocal presentation, defined as the presence of tumor at two or more anatomically separate sites, before the manifestation of disease in sites where sarcomas usually metastasize (e.g., lungs) occurs in about 1% of extremity soft tissue sarcomas [7].

An interesting question is whether these extrapulmonary tumors in fact represent metastatic disease versus sites of synchronous or metachronous primary disease [8]. Tedeschi proposed the concept of a “pluricentric anlage” or “incidental stimulation of undifferentiated mesenchymal cells” due to altered lipid metabolism [10] as an explanation to why patients he observed had multiple lipomatous tumors in fat-bearing soft tissue locations. An analogy has been drawn between this phenomenon and the clinical presentation of multiple subcutaneous nodules in patients with neurofibromatosis [11]. Smith et al. analyzed the genomic rearrangements of *TLS*, *CHOP*, or *EWS* in six patients and confirmed the monoclonal origin of multifocal MRCL [4]. They concluded that this unusual clinical phenomenon most likely represents a pattern of presumably hematogenous metastasis to other soft tissue sites, by tumor cells seemingly incompetent to seed the lungs [4]. Definitive differentiation between metastatic disease and synchronous or metachronous primary disease is elusive and likely will become possible only by molecular biologic analysis of tumor clonal heterogeneity [12].

Prognostic factors of MRCL have been described in several previous studies. Age (>45 years) [6], large tumor size (≥10 cm) [14, 15], percentage of round cell differentiation (≥5%) [4, 15], and presence of tumor necrosis [6] were associated with a poor prognosis. Antonescu et al. also sought to evaluate the potential impact of *TLS-CHOP* fusion transcript structure on clinical outcome in 82 cases of localized MRCL [5]. They concluded that in contrast to some other translocation associated sarcomas, such as Ewing sarcoma, synovial sarcoma, and rhabdomyosarcoma, the molecular variability of *TLS-CHOP* fusion transcript structure does not appear to have a significant impact on clinical outcome in localized MRCL [5]. However, high histological grade (≥5% round cell component), presence of necrosis, and P53 overexpression were independent predictors of unfavorable outcome in localized MRCL [5]. To our knowledge, parameters that influence the pattern of metastases have not been reported to date. Our results suggest that tumor size, histological grade, and implementation of chemotherapy have significant influence on metastatic patterns. Large tumor size and low histological grade were significantly associated with extrapulmonary metastasis. Also, presence of chemotherapy was significantly associated with metastasis, but there is a bias here in that higher rate of chemotherapy was performed on patients with high-grade tumors: 15 of 20 patients (75%) compared to patients with low-grade tumors: 14 of 38 patients (37%). Chemotherapy was not effective in reducing disease-free survival rate.

In conclusion, extrapulmonary metastasis was observed in 14% of the cases with MRCL. The bone was the most common location for extrapulmonary metastasis. Large tumor size and low histological grade were significantly associated with extrapulmonary metastasis. These findings might lead to new diagnostic and treatment options for MRCL.

## Figures and Tables

**Table 1:** Influence of pathologic variables on metastatic pattern.

Parameters	Total	Absence of metastasis	Extrapulmonary metastasis	Pulmonary metastasis	*P* value
Patients (No.)	58	47	8	3	
Age at diagnosis					
Median age (range)	46 (18–80)	44 (18–80)	51 (37–66)	58 (32–72)	NS
≤45	28	24	3	1	0.699529
>45	30	23	5	2	

Gender					
Male	33	27	3	3	NS
Female	25	20	5	0	0.127731

Tumor size					
Median size (range)	9.3 (1.2–25)	8.7 (1.2–20)	15.5 (10–25)	9 (4–14)	*P* < 0.05
≥10 cm	31	29	0	2	0.027573
≥10 cm	19	14	4	1	
unknown	8	4	4	0	

Tumor depth					
Superficial	13	11	1	2	NS
Deep	39	32	5	1	0.351308
Unknown	6	4	2	0	

Tumor site					
Extremity	48	41	5	2	NS
Trunk	10	6	3	1	0.29337

Histological grade					
Low grade	38	34	4	0	*P* < 0.05
High grade	20	13	4	3	0.026703

**Table 2:** Influence of treatment variables on metastatic patterns.

Parameters	Total	Absence of metastasis	Extrapulmonary metastasis	Pulmonary metastasis	*P* value
Patients (No.)	58	47	8	3	
Surgical margin					
Intralesional	1	1	0	0	
Marginal	16	12	3	1	NS
Wide	40	34	4	2	0.700905

Radiotherapy					
Yes	13	9	3	1	NS
No	45	38	5	2	0.550657

Chemotherapy					
Yes	29	20	6	3	*P* < 0.05
No	29	27	2	0	0.0037966

Local recurrence					
Yes	5	3	1	1	NS
No	53	44	7	2	0.51357

Disease free					
Yes	50		4	0	*P* < 0.001
No	8		4	3	4.40*E* − 05

**Table 3:** Comparison between previous and current study.

Year	Author	No. of cases	Metastatic rate	Extrapulmonary metastatic rate	Pulmonary metastatic rate	Common sites of the extrapulmonary metastases
2007	Schwab JH	230	31%	17%	14%	Bone, Soft tissue, Abdomen
1999	Spillane AJ	50	—	20%	—	Abdomen, Retroperitoneum
1997	Pearlstone DB	102	32%	30%	2%	Retroperitoneum, Chest wall
1996	Kilpatrick SE	95	35%	—	—	Retroperitoneum, Abdomen, Chest wall
2010	Current study	58	19%	14%	5%	Bone, Soft tissue
